# Self-Supervised and Zero-Shot Learning in Multi-Modal Raman Light Sheet Microscopy

**DOI:** 10.3390/s24248143

**Published:** 2024-12-20

**Authors:** Pooja Kumari, Johann Kern, Matthias Raedle

**Affiliations:** 1 CeMOS Research and Transfer Center, Mannheim University of Applied Sciences, 68163 Mannheim, Germany; m.raedle@hs-mannheim.de; 2 Universitätsklinikum Mannheim, Universität Heidelberg, 68167 Mannheim, Germany; johann.kern@medma.uni-heidelberg.de

**Keywords:** deep learning, unsupervised learning, zero-shot learning, self-supervised learning, super-resolution, denoising, light sheet microscopy, Raman scattering, Rayleigh scattering, fluorescence, spheroid, multi-mode

## Abstract

Advancements in Raman light sheet microscopy have provided a powerful, non-invasive, marker-free method for imaging complex 3D biological structures, such as cell cultures and spheroids. By combining 3D tomograms made by Rayleigh scattering, Raman scattering, and fluorescence detection, this modality captures complementary spatial and molecular data, critical for biomedical research, histology, and drug discovery. Despite its capabilities, Raman light sheet microscopy faces inherent limitations, including low signal intensity, high noise levels, and restricted spatial resolution, which impede the visualization of fine subcellular structures. Traditional enhancement techniques like Fourier transform filtering and spectral unmixing require extensive preprocessing and often introduce artifacts. More recently, deep learning techniques, which have shown great promise in enhancing image quality, face their own limitations. Specifically, conventional deep learning models require large quantities of high-quality, manually labeled training data for effective denoising and super-resolution tasks, which is challenging to obtain in multi-modal microscopy. In this study, we address these limitations by exploring advanced zero-shot and self-supervised learning approaches, such as ZS-DeconvNet, Noise2Noise, Noise2Void, Deep Image Prior (DIP), and Self2Self, which enhance image quality without the need for labeled and large datasets. This study offers a comparative evaluation of zero-shot and self-supervised learning methods, evaluating their effectiveness in denoising, resolution enhancement, and preserving biological structures in multi-modal Raman light sheet microscopic images. Our results demonstrate significant improvements in image clarity, offering a reliable solution for visualizing complex biological systems. These methods establish the way for future advancements in high-resolution imaging, with broad potential for enhancing biomedical research and discovery.

## 1. Introduction

Biomedical imaging plays a crucial role in advancing our understanding of complex biological systems, particularly three-dimensional (3D) structures such as cell cultures, spheroids, and organoids. These 3D structures have become fundamental models in drug discovery, cancer research, and histology, offering insights into tissue organization and cellular interactions. Traditional imaging techniques, such as fluorescence microscopy and electron microscopy, have long been used to visualize these structures. However, many of these methods require invasive sample preparation or the use of external labeling agents, which can introduce artifacts and affect the biological systems under observation.

The major advantages of 3D cell cultures over 2D cell cultures are that cell cultures in free three-dimensional space grow much more similarly to real organs and react to pharmaceuticals than flat cell cultures. The use of 3D cell cultures thus shows a strict way to avoid animal experiments to an ever greater extent. As a result, there is a growing need for imaging techniques that allow for non-invasive, label-free visualization of biological samples in their native states [1,2,3].

Raman light sheet microscopy has emerged as a powerful, non-invasive, and label-free imaging modality that enables the study of 3D biological structures without altering the biological samples. This technique combines Rayleigh scattering, Raman scattering, and fluorescence detection, offering complementary spatial and molecular information about cellular architecture and interactions. The multi-modal approach of Raman light sheet microscopy provides a unique advantage by capturing rich data from biological systems, including high-resolution images of both spatial organization and molecular composition. The individual image modes contrast different information of the cell structure without using dye markers: image-based elastic scattering shows the arrangement of cell walls and nuclei; fluorescence imaging indicates, e.g., the spatial distribution of collagen or diffused pharmaceuticals; and Raman imaging shows, e.g., the distribution of hydrocarbons or amino acids, or even water. The label-free nature of this technique minimizes the risk of sample perturbation, making it ideal for studying delicate biological structures like spheroids and organoids in their natural state [4,5]. Even growing processes in living cells could be observed.

Despite its advantages, Raman light sheet microscopy faces several technical challenges, particularly related to image noise and resolution limitations. Raman scattering, although valuable for providing molecular information, generates extremely weak signals that are prone to noise. This, coupled with low signal intensity, often limits the achievable resolution, making it difficult to capture fine subcellular details in 3D samples [6,7]. Traditional methods, such as deconvolution and spectral unmixing, have been employed to enhance image quality, but these techniques often require complex preprocessing steps or rely on large amounts of labeled data, which can be impractical in real-time imaging [8,9].

Recent advances in machine learning, particularly deep learning techniques, offer promising solutions to overcome these limitations. While deep learning has been successfully applied to enhance resolution and reduce noise in medical imaging, these methods typically require extensive amounts of high-quality, labeled data for training. However, for applications like Raman light sheet microscopy, obtaining such labeled datasets can be impractical. This has led to the exploration of zero-shot and self-supervised learning approaches, which can enhance image quality without the need for labeled data [10,11].

Techniques such as Noise2Void, Deep Image Prior (DIP), and ZS-DeconvNet represent state-of-the-art approaches in zero-shot and self-supervised learning. These methods operate by leveraging the inherent structure and statistical properties of the data itself, enabling them to perform denoising and super-resolution without labeled training datasets. Their ability to enhance image clarity and resolution in an unsupervised manner makes them particularly well suited for multi-modal Raman light sheet microscopy [1,12,13].

This paper presents a comprehensive comparative evaluation of zero-shot and self-supervised learning algorithms for denoising and super-resolution in multi-modal Raman light sheet microscopy. By systematically evaluating these algorithms across different imaging modalities, we aim to identify the most effective techniques for improving image quality while preserving biological fidelity. This work aims to advance the field of high-resolution biomedical imaging and facilitate more accurate visualization of complex biological systems [1,10,14].

## 2. Materials and Methods

### 2.1. Biological Samples

Biological samples, including 3D spheroids and cell cultures, were prepared using established protocols to ensure optimal imaging conditions while maintaining cellular integrity. Spheroids were embedded in a low-scattering hydrogel matrix, providing optical transparency, and preserving physiological conditions during imaging. This method minimized light scattering, ensuring high-quality imaging while maintaining an environment conducive to cellular function.

For this study, spheroids were generated from two HPV-negative head and neck squamous cell carcinoma (HNSCC) cell lines: UMSCC-11B, derived from laryngeal carcinoma, and UMSCC-14C, from oral cavity carcinoma. Both cell lines were cultured in Eagle’s minimum essential medium (EMEM) supplemented with 10% fetal bovine serum (FBS) and 1% Penicillin/Streptomycin. Cultures were maintained at 37 °C in a humidified 5% CO_2_ atmosphere. Cells were detached using Trypsin/EDTA, counted using a Neubauer hemocytometer, and seeded into ultra-low attachment (ULA) 96-well plates at densities of 2.5 × 10^4^ or 5 × 10^4^ cells per well to generate spheroids. These spheroids were cultured for up to eight days, with media changes on days 3, 5, and 8, reaching a diameter of 300–400 µm.

To investigate drug treatment effects, spheroids were treated with cisplatin (50 µM) on day 4 of culture, while control spheroids were treated with DMSO. After 72 h of incubation, both treated and untreated spheroids were fixed in 4% formalin to preserve their structural integrity for subsequent imaging. Samples were mounted in a 3D-printed hydrogel carrier designed for optimal alignment with the light sheet and detection objective, allowing multi-view imaging at 37 °C with 5% CO_2_ and ensuring sample viability during extended imaging sessions.

### 2.2. Raman Light Sheet Microscope

**Figure 1:**
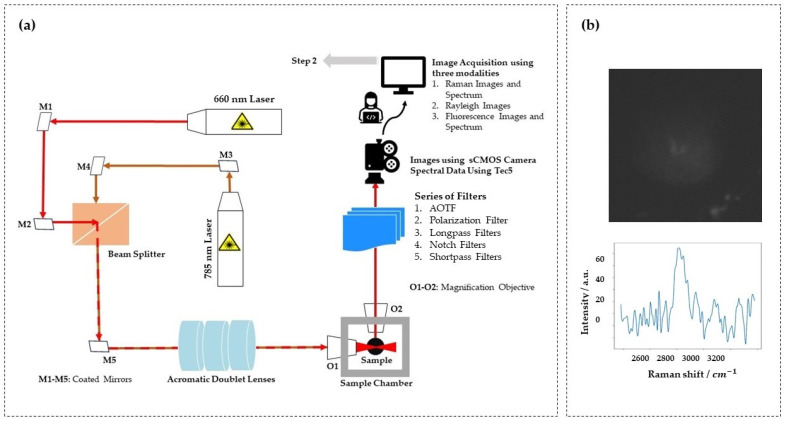
(**a**) Schematic of the multi-modal Raman light sheet microscope and (**b**) Raman image and spectral data acquired using a multi-modal Raman light sheet microscope with a 660 nm excitation laser and an acousto-optic tunable filter (AOTF) set at 817 nm.

The Raman light sheet microscope developed in this study integrates Rayleigh scattering, fluorescence, and Raman scattering modalities into a single, high-precision platform. The system utilizes dual-laser architecture, featuring a 660 nm and a 785 nm continuous wave laser, coaxially aligned through a series of broadband coated mirrors (M1-M5) and a beam splitter. These beams are then passed through achromatic doublet lenses to generate a well-defined, static light sheet that illuminates the sample chamber. (Figure 1a).

The illumination optics generate a vertically oriented light sheet in the sample chamber, while the orthogonally positioned detection optics collect scattered or emitted photons. The axial resolution of the light sheet, defined by the beam waist, was determined to be approximately 8 µm for both the 785 nm and 660 nm lasers. This was measured using a BP209-VIS/M Scanning-Slit Optical Beam Profiler (Thorlabs Inc., Newton, MA, USA), ensuring uniform illumination and minimal scattering artifacts during imaging. This configuration supports precise optical slicing with a step size of 10 µm along the optical axis, crucial for capturing high-resolution subcellular structures in sequential imaging. The system’s effective imaging field of view, calculated as 635 µm × 635 µm, accommodates spheroid samples with diameters up to this size, enabling complete optical sectioning for various experimental conditions. These parameters ensure high fidelity in multi-modal Raman imaging, particularly when paired with robust sample positioning for accurate alignment during sequential acquisitions. At the detection interface, a sCMOS camera records the emitted or scattered light after it passes through a configurable filter assembly. The filter set includes an acousto-optic tunable filter (AOTF), polarization filters, and a combination of longpass, notch, and shortpass filters. The AOTF enables precise spectral selection, allowing for fine-tuning of the transmitted wavelengths for each modality. This flexibility is critical for switching between the Rayleigh, Raman, and fluorescence modes without physically altering the optical setup. The polarization filters further improve contrast by rejecting unwanted light, enhancing the efficiency of inelastic scattering detection.

The detection system comprises a sCMOS camera paired with a modular filter assembly that includes an acousto-optic tunable filter (AOTF), polarization filters, and various longpass, notch, and shortpass filters. This modular system ensures that specific wavelengths are selected for each modality without physical realignment, enabling seamless transition between imaging modes. A multi-axis stage ensures precise sample positioning, with submicron resolution along the X, Y, and Z axes, and rotational control. This fine control is essential for maintaining stable and consistent imaging, particularly when working with 3D biological samples such as spheroids.

Figure 1b presents the Raman image and spectral data acquired using a 660 nm laser for excitation and an acousto-optic tunable filter (AOTF) for detection, centered at a wavelength of 817 nm.

### 2.3. Image Acquisition and Data Management

The multi-modal imaging system allows for comprehensive data acquisition using Rayleigh, fluorescence, and Raman modalities. For Rayleigh scattering, imaging was performed with a 785 nm laser at 1 mW power and 100 ms exposure, with an AOTF wavelength of 775 nm. Additionally, Rayleigh imaging at 660 nm was conducted under the same conditions, but with an AOTF wavelength of 650 nm. This enabled high-contrast Rayleigh data collection with minimal interference from other signals (Table 1).

For fluorescence imaging, a 660 nm laser was employed with a power output of 130 mW and an exposure time of 5000 ms, with the AOTF adjusted to 694 nm. This longer exposure time was critical to accommodate the inherently weaker fluorescence signals. Raman spectroscopy was conducted using the same 660 nm laser at 130 mW, with an exposure time of 5000 ms and an AOTF wavelength set to 817 nm, ensuring an optimal signal-to-noise ratio in capturing Raman spectra.

Data acquisition was synchronized across all modalities, and each dataset was meticulously archived with detailed metadata, including laser power, wavelength, exposure time, and filter settings. This approach ensured reproducibility and traceability, allowing for comparative evaluation across different modalities. The system’s capability to rapidly switch between imaging modes via the tunable filter set facilitated efficient data collection, significantly reducing downtime between modality transitions.

By integrating these three imaging modalities into a single experimental setup, the Raman light sheet microscope provided a robust platform for high-resolution, multi-modal data acquisition. This comprehensive data management strategy further ensured that collected datasets could be efficiently processed and analyzed for detailed insights into the samples.

### 2.4. Image Processing and Enhancement Using Deep Learning

#### 2.4.1. Preprocessing

**Figure 2:**
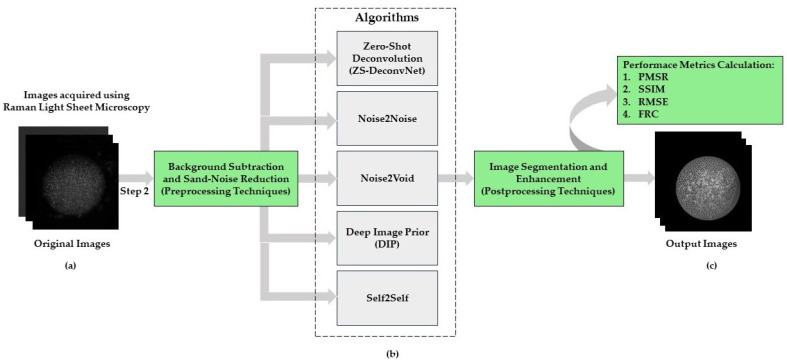
(**a**) Original images acquired using Multi-Modal Raman Light Sheet Microscopy (**b**) Implementation of zero-shot and self-supervised learning algorithms (ZS-DeconvNet, Noise2Noise, Noise2Void, DIP and Self2Self) on Original Images after Preprocessing Techniques (**c**) Denoised Output Images evaluated using metrics (PSNR, SSIM, RMSE, FRC).

Before applying zero-shot and self-supervised learning algorithms, multi-modal original images (Figure 2a) undergo background subtraction to eliminate unwanted signals and sand-noise reduction using filters like Gaussian or median filtering. These steps ensure clean, noise-reduced images, crucial for improving the performance and accuracy of the subsequent learning models.

#### 2.4.2. Zero-Shot and Self-Supervised Learning Algorithms

To address the inherent blurring and noise challenges in multi-modal Raman light sheet microscopy, we applied the zero-shot deconvolution network (ZS-DeconvNet), an unsupervised deep learning model designed to perform deconvolution directly on noisy images without the need for labeled training datasets. This approach allowed us to significantly enhance the resolution of microscopy images and recover finer subcellular structures that are otherwise obscured by system-induced blurring. In this study, we compared a series of advanced self-supervised and zero-shot learning methods aimed at denoising and enhancing resolution in multi-modal Raman light sheet microscopy. Each method was selected based on its ability to improve image clarity without requiring large, labeled datasets—an important consideration given the difficulty of acquiring clean, high-resolution ground truth images in microscopy. The following sections describe the methodologies employed: Zero-Shot Deconvolution Network (ZS-DeconvNet), Noise2Noise, Noise2Void, Deep Image Prior (DIP), and Self2Self (Figure 2b).

##### Zero-Shot Deconvolution Network (ZS-DeconvNet)

The zero-shot deconvolution network (ZS-DeconvNet) is a deep learning model designed to perform image deconvolution directly from noisy and corrupted images without the need for clean reference images during training. Operating in a zero-shot manner, ZS-DeconvNet adapts to each specific image stack during the deconvolution process, making it highly suitable for enhancing microscopy images, where acquiring high-quality, labeled training data is challenging or impractical. ZS-DeconvNet is particularly effective in applications such as multi-modal Raman light sheet microscopy, where images are frequently degraded by system-induced blur and noise. The algorithm learns to reverse the blurring process directly from the noisy input, recovering sharp details while preserving the biological structures. Specifically, it addresses the challenge of image blurring caused by the system’s point-spread function (PSF) in multi-modal microscopy. As a fully convolutional neural network (CNN), ZS-DeconvNet operates within a zero-shot learning framework, allowing the model to learn and reverse the convolutional effects of the PSF from the observed image data, without relying on any external clean datasets [15,16,17].

Mathematical Formulation:

The observed microscopy image Y can be modeled as the convolution of the latent sharp image X with the point-spread function (PSF) h, combined with noise n:

(1) Y=h∗X+n

ZS-DeconvNet optimizes the network parameters θ by minimizing the loss between the convolved network output $f_{\theta }(h*Y)$ and the noisy observation Y:

(2) θ^=argmin θ⁡Y−fθ(h∗Y)2

By minimizing this loss, the network learns to perform deconvolution and recover the underlying image structure.

Network Architecture:

The architecture consists of an encoder–decoder structure with skip connections to retain spatial details. The encoder compresses the input image, while the decoder restores the image resolution:

1. Encoder: Three layers of Conv2D, BatchNormalization, ReLU activation, and MaxPooling2D progressively downsample the image.
2. Decoder: The decoder mirrors the encoder with UpSampling2D layers followed by concatenation with the corresponding encoder layers, allowing detailed feature recovery.

Training Process:

ZS-DeconvNet was trained using pairs of corrupted images generated by applying random Gaussian noise to the input. The model was optimized using Adam for 100 epochs with mean squared error (MSE) as the loss function. The results demonstrated significant improvements in image sharpness and noise reduction, particularly in resolving subcellular structures [1,17,18].

##### Noise2Noise

Noise2Noise is a self-supervised learning algorithm designed for denoising tasks where clean reference images are unavailable. Instead of learning from noisy–clean pairs, Noise2Noise uses pairs of noisy images to learn to suppress noise while preserving the underlying image content [19].

Mathematical Formulation:

Noise2Noise operates under the assumption that noise present in the image is independent across acquisitions, but the underlying clean image remains constant. Given two noisy observations $Y_{1}$ and $Y_{2}$ represent two noisy observations of the same underlying clean image X, the model learns a mapping $f_{\theta }$ to predict one noisy observation from another:

(3) $$Y_{1}=X+n_{1}$$

(4) $$Y_{2}=X+n_{2}$$

where $n_{1}$ and $n_{2}$ are independent noise realizations and $f_{\theta }$ is the convolutional neural network (CNN) with learnable weights θ. The network $f_{\theta }$ is trained to predict $Y_{2}$ from $Y_{1}$ with the objective of minimizing the mean squared error (MSE) between the predicted and observed noisy images:

(5) $$L\left(\theta \right)=\frac{1}{N}\sum_{i=1}^{N}|{\left|f_{\theta }\left(Y_{1}\right)-Y_{2}|\right|}^{2}$$

By optimizing this loss, the network learns to denoise the input image by recovering the shared structure between the noisy pairs while ignoring the noise.

Network Architecture:

The model used a U-Net-like encoder–decoder structure, similar to ZS-DeconvNet, but with a focus on learning from noisy image pairs rather than reconstructing from blurred images. The same Conv2D, BatchNormalization, and ReLU activation layers were used in both the encoder and decoder sections, with skip connections to ensure detail preservation.

Training Process:

Training was conducted on pairs of noisy images, with random Gaussian noise added to simulate real-world noise in microscopy. The model was trained for 100 epochs using the Adam optimizer and MSE loss, achieving high-quality denoising without requiring clean training data [20].

##### Noise2Void

Noise2Void is a self-supervised learning approach designed specifically for denoising tasks in the absence of paired noisy–clean image data. Unlike traditional supervised methods, Noise2Void learns to restore clean images from a single noisy image by exploiting the local structure of the image itself. This is achieved by predicting pixel values based on the context provided by neighboring pixels, masking the central pixel during training to prevent the model from directly learning the noise pattern [21].

Mathematical Formulation:

Let Y represent the observed noisy image and X the underlying clean image, with the relationship modeled as:

(6) Y=X+n

where n is the noise. Noise2Void operates by applying a blind-spot strategy, where the central pixel in the receptive field is masked, and the network is trained to predict the value of the masked pixel using the surrounding pixels as context. The loss function for training is defined as:

(7) $$L\left(\theta \right)=\frac{1}{N}\sum_{i=1}^{N}|{\left|f_{\theta }\left(Y_{\backslash i}\right)-Y_{i}|\right|}^{2}$$

Here, $Y_{\backslash i}$ represents the image with pixel i masked, and $f_{\theta }\left(Y_{\backslash i}\right)$ is the CNN’s prediction for the masked pixel value. By minimizing the difference between the predicted and actual pixel values, the model learns to denoise the image while preserving structural details.

Network Architecture:

Noise2Void used a U-Net-like architecture, similar to the one used for Noise2Noise. The encoder–decoder architecture was designed to capture both local and global context from the input image, allowing the network to predict the missing pixel values.

Training Process:

Blind-spot masking was applied to the input images during training, ensuring that the model never learns from the pixel it is supposed to predict. This forces the network to rely on surrounding context, making it effective for noise suppression in microscopy images. The model was trained for 1000 epochs using the Adam optimizer with early stopping to prevent overfitting [22,23].

##### Deep Image Prior (DIP)

The deep image prior (DIP) is a self-supervised learning approach that leverages the structure of convolutional neural networks (CNNs) as an implicit regularizer for image restoration tasks, such as denoising and super-resolution, without the need for pretrained models or large labeled datasets. Unlike traditional deep learning models, DIP directly trains on a single noisy image, using the architecture of the CNN itself to impose regularization on the output. This makes DIP particularly useful for microscopy applications, where obtaining clean, labeled ground-truth images is difficult [24].

Mathematical Formulation:

Given a noisy observation Y, DIP aims to recover the underlying clean image X by optimizing the CNN $f_{\theta }\left(z\right)$, where z is a fixed random input. The optimization objective is expressed as:

(8) θ^=argmin θ⁡fθz−Y2

Here, $f_{\theta }\left(z\right)$ is the CNN with learnable weights θ, and z is typically a fixed random noise input. The key innovation of DIP is that the network is not pretrained on external datasets; rather, it learns to denoise the image directly during the optimization process. The network’s architecture naturally regularizes the output by capturing image priors such as smoothness and continuity, which are inherent in most natural images.

Network Architecture:

The architecture for DIP is an encoder–decoder CNN similar to other methods described but uses random noise as input. Skip connections are used to retain fine details, and the decoder reconstructs the image from its compressed representation. The final output is generated by applying a sigmoid activation to constrain the pixel values between 0 and 1.

Training Process:

The model was trained directly on the noisy microscopy image, using early stopping to prevent overfitting. Training typically converged after 1000 epochs, yielding results that improved both image clarity and structural preservation [25].

DIP achieves denoising by regularizing the optimization process. As training progresses, the network gradually captures the underlying image structure, and denoising occurs as a natural outcome of the training process. The network converges to a solution where the output $f_{\theta }\left(z\right)$ represents a denoised version of the input image.

##### Self2Self

Self2Self is a self-supervised denoising technique that employs dropout as a form of regularization, allowing it to learn directly from noisy images without requiring clean or paired data. Unlike other denoising algorithms that rely on multiple noisy images or clean references, Self2Self is designed to operate on a single noisy image, using dropout to mask out pixels and learning to predict missing values based on the remaining context. This makes Self2Self highly effective in applications where acquiring multiple noisy samples or clean labels is not feasible, such as multi-modal Raman light sheet microscopy [26,27].

Mathematical Formulation:

Given a noisy observation Y, which is modeled as:

(9) Y=X+n

where X is the latent clean image and n is the noise, Self2Self applies random dropout to the input image, masking a portion of pixels during training. The objective is to train the network to reconstruct the clean image by predicting the dropped pixels using the remaining visible ones. The dropout introduces randomness, which acts as a form of regularization, preventing the network from overfitting to the noise.

The training loss function is defined as:

(10) $$L\left(\theta \right)=\frac{1}{N}\sum_{i=1}^{N}|{\left|f_{\theta }\left(Y_{dropout}\right)-Y|\right|}^{2}$$

Here, $f_{\theta }\left(Y_{dropout}\right)$ is the network’s prediction for the masked pixels and Y is the noisy input image. The dropout mask ensures that the network is forced to learn useful features of the underlying clean image rather than overfitting to the noise.

Network Architecture:

The Self2Self network architecture is similar to DIP but incorporates dropout layers to mask a portion of the input pixels during training. This forces the network to learn useful features from the unmasked pixels while avoiding overfitting to noise.

Training Process:

Self2Self was trained with random dropout applied to the noisy image during each training step. The model was optimized using Adam for 1000 epochs, and early stopping was applied to halt training when the loss stopped improving. Self2Self demonstrated effective denoising while preserving key subcellular structures [28].

#### 2.4.3. Model Training and Implementation

All models were implemented using the TensorFlow deep learning framework. Training and inference were conducted on a high-performance computing system equipped with NVIDIA A100 GPUs, enabling fast, parallelized processing of the large multi-modal microscopy datasets.

Training Details

Each algorithm was trained on the same set of input images, consisting of noisy or corrupted microscopy data. Hyperparameters, including the learning rate and batch size, were fine-tuned to optimize each model’s performance. The following key hyperparameters and parameters were used across all models, as shown in Table 1.

Each model was trained using these parameters and hyperparameters until convergence, defined as no improvement in the loss function for 10 consecutive epochs. For models like ZS-DeconvNet and Noise2Noise, 100 epochs were sufficient due to their efficiency in learning image structures from noisy pairs. Due to the large size of the 3D microscopy image stacks, a batch size of 1 was used across all models. This allowed efficient memory usage on the GPUs while maintaining high-performance processing, particularly for models that require the handling of large, high-dimensional data. These parameters were selected same for fair performance comparison.

#### 2.4.4. Evaluation Metrics

The performance of the denoising and image enhancement algorithms was evaluated using a series of quantitative metrics to assess the quality of the output images, particularly focusing on the preservation of biological structures and overall noise reduction.

##### Peak Signal-to-Noise Ratio (PSNR)

PSNR was calculated to measure the quality of the denoised images relative to the original noisy inputs. A higher PSNR value indicates better noise suppression, with less distortion introduced during the image restoration process. PSNR is defined as [28,29]:

(11) $$PSNR={10\times \log}_{10}\left(\frac{{MAX}_{I}^{2}}{MSE}\right)$$

where ${MAX}_{I}$ is the maximum possible pixel value of the image and MSE is the mean squared error between the original and processed image. Higher PSNR values suggest better noise suppression, meaning the deblurred image is closer to the clean image, which is ideal for microscopy, where noise can obscure fine details of subcellular structures. PSNR values above 30 dB generally indicate good image quality with significant noise reduction. For microscopy images, values between 30 and 50 dB are common, with values closer to 50 dB indicating near-perfect noise reduction and high-quality image restoration [27,30].

##### Structural Similarity Index (SSIM)

The structural similarity index (SSIM) is a metric designed to evaluate the perceived quality of an image by comparing luminance, contrast, and structural information between two images. SSIM ensures that the structural integrity of the deblurred images is preserved, especially critical for maintaining the accuracy of biological structures such as cells and organelles [31].

SSIM is calculated as:

(12) $$SSIM\left(x,y\right)=\frac{\left(2\mu_{x}\mu_{y}+C_{1})({2\sigma }_{xy}+C_{2}\right)}{\left(\mu_{x}^{2}+\mu_{y}^{2}+C_{1})(\sigma_{x}^{2}+\sigma_{y}^{2}+C_{2}\right)}$$

where:

$\mu_{x}$ and $\mu_{y}$ are the mean intensities of images x and y.

$\sigma_{x}^{2}$ and $\sigma_{y}^{2}$ are the variances of the images x and y, respectively.

$\sigma_{xy}$ is the covariance between the images.

$C_{1}$ and $C_{2}$ are constants to stabilize the division when the denominator is close to zero.

SSIM values above 0.85 indicate that the structural content of the image is well preserved, and there is minimal distortion. Ideal SSIM values for high-quality biological microscopy images range between 0.90 and 0.99, indicating that the structural similarity between the noisy and denoised images is high [19,26].

##### Root Mean Squared Error (RMSE)

Root mean squared error (RMSE) measures the pixel-wise error between the noisy input and the denoised output. Lower RMSE values indicate better performance, with fewer deviations between the noisy and denoised images. RMSE is particularly useful for quantifying how accurately the algorithm has reconstructed the image from noisy input [21].

(13) $$RMSE=\sqrt{\frac{1}{N}\sum_{i=1}^{N}{\left(I_{noisy,i}-I_{denoised,i}\right)}^{2}}$$

where:

N is the total number of pixels in the image.

$I_{original}$ and $I_{deblurred}$ are the pixel intensities in the original and deblurred images, respectively.

Lower RMSE values indicate better performance. In microscopy, RMSE values below 0.10 are preferred, with values closer to 0.01–0.05 representing excellent noise reduction and minimal pixel-wise error [25,32].

##### Fourier Ring Correlation (FRC) Analysis

Fourier ring correlation (FRC) is used to assess how well the spatial frequency components of the denoised image match those of the original noisy image. FRC is essential for evaluating the retention of high-frequency information, which corresponds to the sharpness and fine details in the image [24,25].

(14) $$FRC=\frac{\sum_{i\epsilon R(f)}{FFT}_{1}\left(i\right)\cdot \bar{{FFT}_{2}\left(i\right)}}{\sqrt{\sum_{i\epsilon R(f)}{\left|{FFT}_{1}\left(i\right)\right|}^{2}\cdot \sum_{i\epsilon R(f)}{\left|{FFT}_{1}\left(i\right)\right|}^{2}}}$$

where:

${FFT}_{1}$ and ${FFT}_{2}$ are the Fourier transforms of the original and denoised images.

R(f) represents the pixels corresponding to the frequency f.

The numerator measures the cross-power spectrum between the two images, and the denominator normalizes it by accounting for the energy of each image.

Higher FRC values indicate that the denoising algorithm has preserved high-frequency information, which is indicative of maintaining sharpness and fine details in the images. FRC values closer to 1 indicate better retention of structural details at high spatial frequencies. Preferred values typically range between 0.7 and 1.0, with higher values indicating excellent resolution and detail preservation in the denoised images [23,33].

#### 2.4.5. Postprocessing

After applying zero-shot and self-supervised learning algorithms, postprocessing techniques such as image segmentation and enhancement are employed to further refine the output. Image segmentation isolates key regions of interest within the image, while enhancement improves visual clarity and contrast, highlighting critical features for evaluation. These steps ensure that the final images are optimized for interpretation and subsequent quantitative evaluation using PSNR, SSIM, RMSE, and FRC.

## 3. Results

We evaluated five zero-shot and self-supervised learning models—ZS-DeconvNet, Noise2Noise, Noise2Void, Deep Image Prior (DIP), and Self2Self—across four distinct multi-modal Raman light sheet microscopy modalities. The results were analyzed based on quantitative metrics, including PSNR, SSIM, RMSE, and FRC, combined with detailed visual comparisons.

### 3.1. Modality 1: Laser: 785 nm; Rayleigh Scattering; AOTF: 775 nm; Sample Type: 14C (Section 2.1)

In the first modality, Rayleigh scattering was imaged using a 785 nm laser, with scattered light filtered through an acousto-optic tunable filter (AOTF) centered at 775 nm to enhance spectral selectivity and minimize background noise. This setup introduces strong noise in regions with low signal intensity. This setup is used for imaging 14C-untreated samples (refer to Section 2.1), where the goal is to enhance image clarity without losing important structural details.

#### 3.1.1. Image Comparison

**Figure 3:**
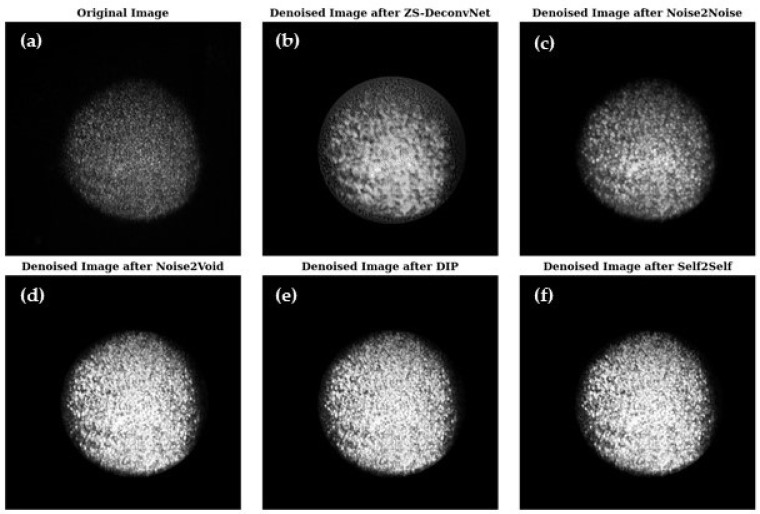
Visual comparison of original and denoised images for all zero-shot and self-supervised learning models for the 785 nm laser and Rayleigh scattering for the untreated 14C samples (refer to Section 2.1).

Figure 3 presents a visual comparison of the denoised images produced by ZS-DeconvNet (Figure 3b), Noise2Noise (Figure 3c), Noise2Void (Figure 3d), DIP (Figure 3e), and Self2Self (Figure 3f), with the original noisy image (Figure 3a) included for reference. Each model exhibits varying levels of noise suppression and structural recovery.

#### 3.1.2. Quantitative Evaluation

**Figure 4:**
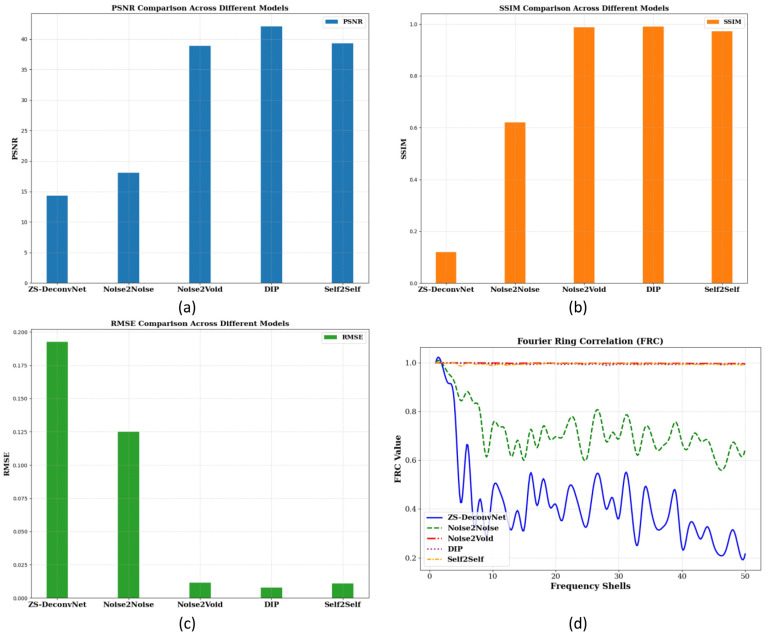
PSNR (**a**), SSIM (**b**), and RMSE (**c**) histograms and FRC curves (**d**) for the 14C-untreated samples and Rayleigh scattering using the 785 nm laser.

To quantitatively assess the model’s performance, we computed PSNR, SSIM, RMSE, and FRC metrics, as shown in Figure 2c. These metrics provide a comprehensive understanding of how well each model balances noise suppression, structural preservation, and high-frequency detail recovery. The PSNR (Figure 4a), SSIM (Figure 4b), RMSE (Figure 4c), and FRC (Figure 4d) analyses for the Rayleigh modality using the 785 nm laser on 14C-untreated samples demonstrate the relative strengths and weaknesses of the tested models. Noise2Void, DIP, and Self2Self emerged as the top performers, achieving the highest PSNR (>40 db), SSIM (close to 1.0), and very low RMSE, maintaining high FRC values, indicating excellent noise suppression and structural preservation. ZS-DeconvNet was moderately effective in noise reduction but struggled to retain structural integrity. Noise2Noise performed better than ZS-DeconvNet with higher PSNR, higher SSIM, lower RMSE, and better information retention, as shown by the FRC curve.

### 3.2. Modality 2: Laser: 785 nm; Fluorescence Scattering; AOTF: 694 nm; Sample Type: 14C (Section 2.1)

In the first modality, fluorescence scattering at 694 nm using a 660 nm laser introduces strong noise in regions with low signal intensity. This setup was used for imaging 14C-untreated samples (refer to Section 2.1), where the goal is to enhance image clarity without losing important structural details.

#### 3.2.1. Image Comparison

**Figure 5:**
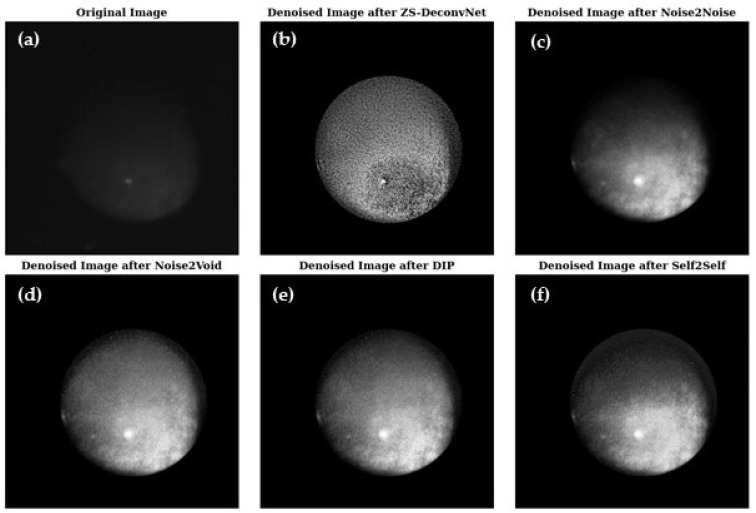
Visual comparison of original and denoised images for all zero-shot and self-supervised learning models for the 660 nm laser and fluorescence scattering for the untreated 14C samples (refer to Section 2.1).

Figure 5 presents a visual comparison of the denoised images produced by ZS-DeconvNet (Figure 5b), Noise2Noise (Figure 5c), Noise2Void (Figure 5d), DIP (Figure 5e), and Self2Self (Figure 5f), with the original noisy image (Figure 5a) included for reference. Each model exhibits varying levels of noise suppression and structural recovery.

#### 3.2.2. Quantitative Evaluation

**Figure 6:**
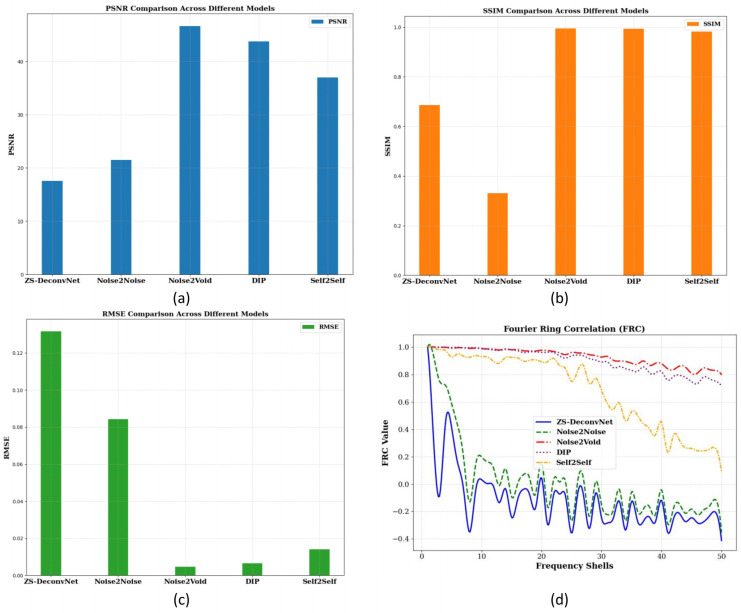
PSNR (**a**), SSIM (**b**), and RMSE (**c**) histograms and FRC curves (**d**) for the 14C-untreated samples and fluorescence scattering using the 785 nm laser.

To quantitatively assess the model’s performance, we computed PSNR, SSIM, RMSE, and FRC metrics, as shown in Figure 2c. These metrics provide a comprehensive understanding of how well each model balances noise suppression, structural preservation, and high-frequency detail recovery. The PSNR (Figure 6a), SSIM (Figure 6b), RMSE (Figure 6c)**,** and FRC (Figure 6d) analyses for the fluorescence modality using the 785 nm laser on 14C-untreated samples demonstrate the relative strengths and weaknesses of the tested models. Noise2Void, DIP, and Self2Self again emerged as the top performers, achieving the highest PSNR (~40 db), SSIM (close to 1.0), and very low RMSE (0.004–0.01), maintaining high FRC values, indicating excellent noise suppression and structural preservation. ZS-DeconvNet performed better in noise reduction but struggled to retain structural integrity. Noise2Noise performed well in noise reduction but failed to retain structural integrity.

### 3.3. Modality 3: Laser: 660 nm; Raman Scattering; AOTF: 817 nm; Sample Type: Treated 11B (Section 2.1)

In this modality, we evaluate Raman signals from 11B-treated samples, which have high noise levels and complex subcellular structures. The challenge lies in suppressing noise while retaining subtle structural details.

#### 3.3.1. Image Comparison

**Figure 7:**
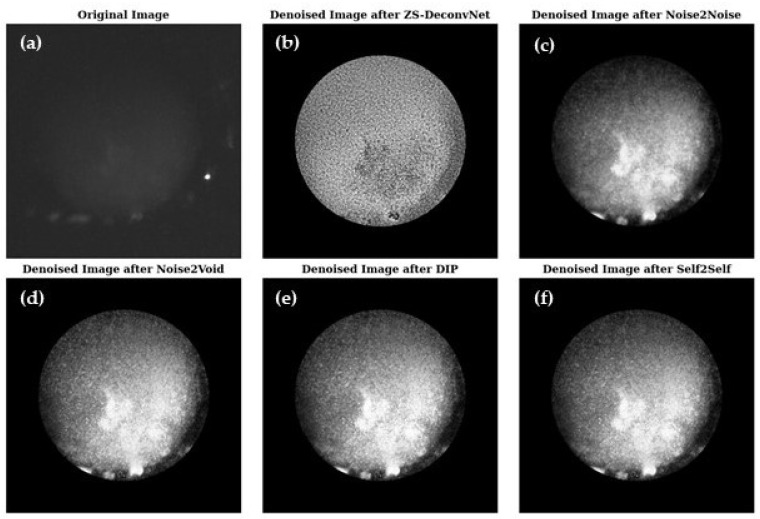
Visual comparison of original and denoised images for all zero-shot and self-supervised learning models for the 660 nm laser and Raman scattering for the treated 11B samples (refer to Section 2.1).

Figure 7 presents a visual comparison of the denoised images produced by ZS-DeconvNet (Figure 7b), Noise2Noise (Figure 7c), Noise2Void (Figure 7d), DIP (Figure 7e), and Self2Self (Figure 7f) with the original noisy image (Figure 7a) included for reference. Each model exhibits varying levels of noise suppression and structural recovery.

#### 3.3.2. Quantitative Evaluation

**Figure 8:**
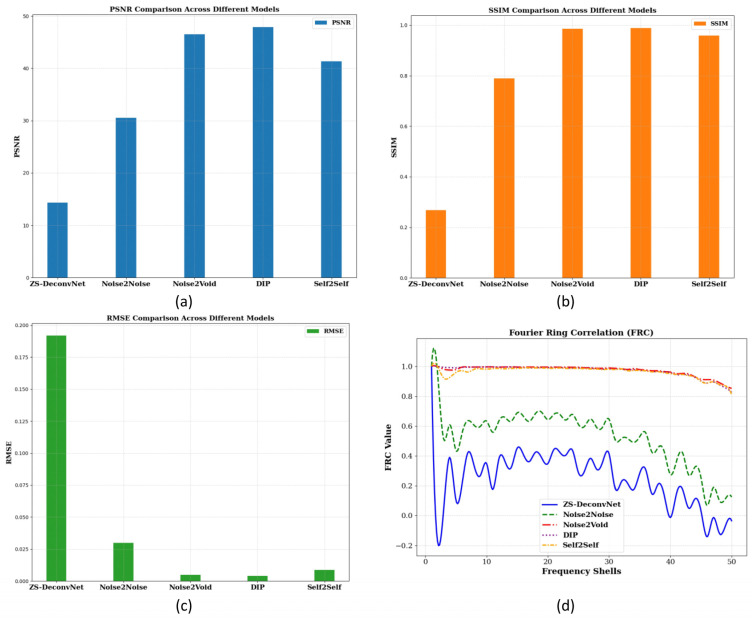
PSNR (**a**), SSIM (**b**), and RMSE (**c**) histograms and FRC curves (**d**) for the 11B-treated samples and Raman scattering using the 660 nm laser.

The PSNR (Figure 8a), SSIM (Figure 8b), RMSE (Figure 8c), and FRC (Figure 8d) analyses for the Raman modality using the 660 nm laser on 11B-treated samples demonstrate the relative strengths and weaknesses of the tested models. ZS-DeconvNet emerged as winner for this modality providing the clearest results—PSNR (~30 db), SSIM (~0.9), and very low RMSE (0.004–0.01), maintaining acceptable FRC values, indicating excellent noise suppression and structural preservation. Noise2Noise, Noise2Void, DIP, and Self2Self also showed improvement in image quality.

### 3.4. Modality 4: Laser: 660 nm; Raman Scattering; AOTF: 817 nm; Sample Type: Untreated 11B (Section 2.1)

In this modality, we evaluate Raman signals from 11B-untreated samples, which have high noise levels and complex subcellular structures. The challenge lies in suppressing noise while retaining subtle structural details.

#### 3.4.1. Image Comparison

**Figure 9:**
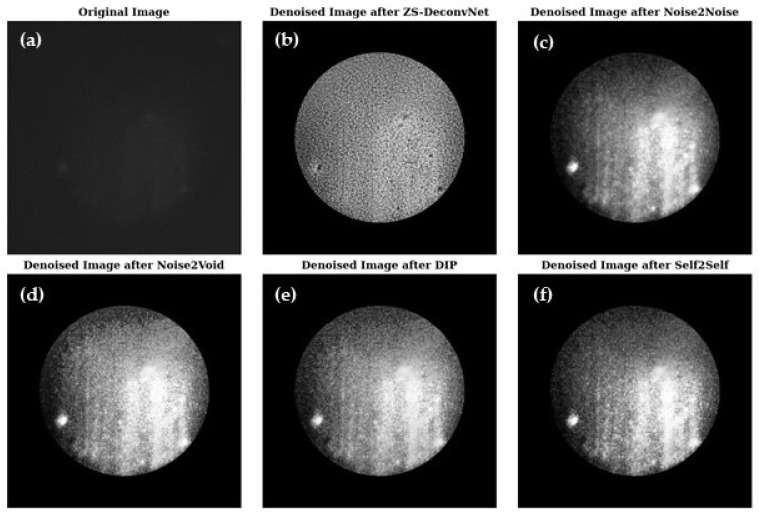
Visual comparison of original and denoised images for all zero-shot and self-supervised learning models for the 660 nm laser and Raman scattering for the untreated 11B samples (refer to Section 2.1).

Figure 9 presents a visual comparison of the denoised images produced by ZS-DeconvNet (Figure 9b), Noise2Noise (Figure 9c), Noise2Void (Figure 9d), DIP (Figure 9e) and Self2Self (Figure 9f) with the original noisy image (Figure 9a) included for reference. Each model exhibits varying levels of noise suppression and structural recovery.

#### 3.4.2. Quantitative Evaluation

**Figure 10:**
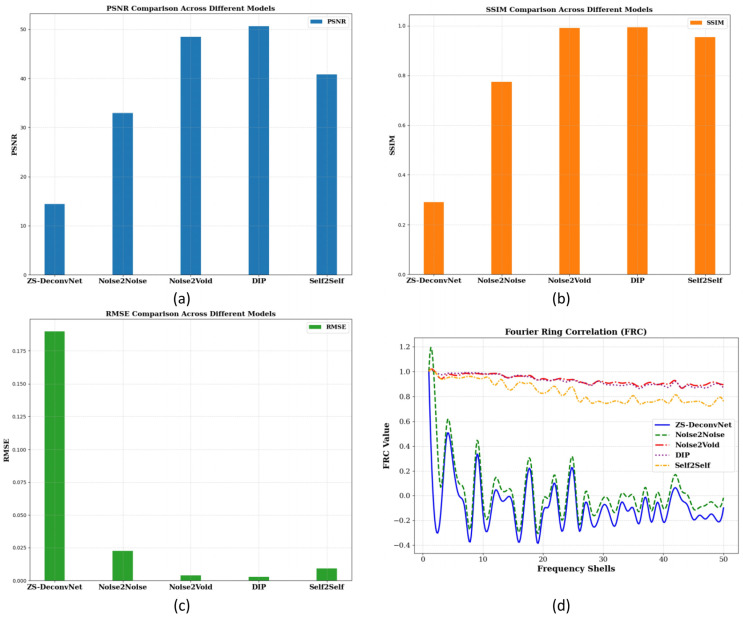
PSNR (**a**), SSIM (**b**), and RMSE (**c**) histograms and FRC curves (**d**) for the 11B-untreated samples and Raman scattering using the 660 nm laser.

Noise2Void, DIP and Self2Self again emerged as the top performers, achieving the highest PSNR (>40 db, Figure 10a), SSIM (close to 1.0, Figure 10b), and very low RMSE (0.004–0.01, Figure 10c), maintaining high FRC values (Figure 10d), indicating excellent noise suppression and structural preservation. ZS-DeconvNet performed better in noise reduction but struggled to retain structural integrity. Noise2Noise performed well in noise reduction but failed to retain structural integrity.

### 3.5. Training Convergence: Loss vs. Epoch Curves

**Figure 11:**
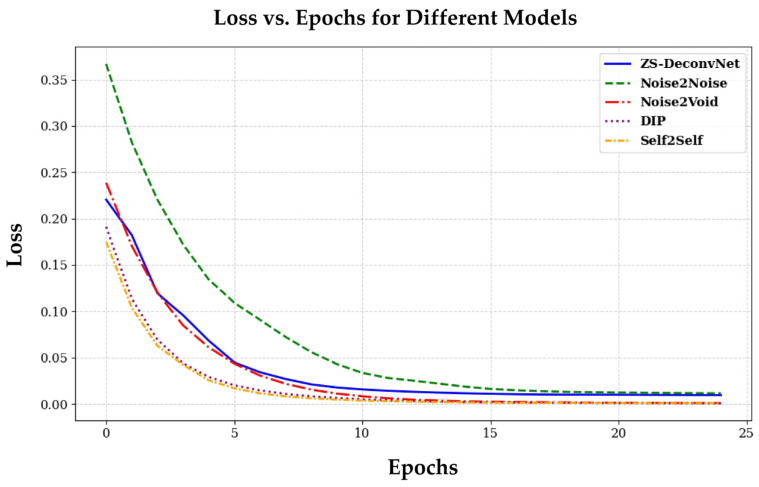
Loss vs. epoch curves for all zero-shot and self-supervised learning algorithms, reflecting overall training performance across all modalities described in Section 3.1.

Figure 11 illustrates the loss vs. epoch curves for the ZS-DeconvNet, Noise2Noise, Noise2Void, DIP, and Self2Self models. The graph shows the first 25 epochs for clarity, although the models were trained for different total epochs: ZS-DeconvNet and Noise2Noise were trained for 100 epochs, while Noise2Void, DIP, and Self2Self were trained for 1000 epochs each (refer to Table 2). All models demonstrate a consistent reduction in loss as the training progresses, reflecting effective optimization across the different architectures. ZS-DeconvNet and Noise2Void exhibit similar convergence profiles, with a steady decline in loss throughout the epochs. Noise2Noise shows a faster reduction in loss during the initial epochs, while DIP and Self2Self exhibit a more gradual but continuous loss minimization over the training period.

These results indicate that all models successfully converge, with steady improvements in loss as the number of epochs increases, signifying effective learning and optimization of the respective models.

## 4. Discussion

In this study, we performed an extensive comparative evaluation of five state-of-the-art zero-shot and self-supervised learning methods for denoising and super-resolution in multi-modal Raman light sheet microscopy. The models—ZS-DeconvNet, Noise2Noise, Noise2Void, Deep Image Prior (DIP), and Self2Self—were evaluated across key quantitative metrics, including PSNR, SSIM, RMSE, and FRC. Across all modalities tested, we observed consistent trends in performance with DIP, Noise2Void, and Self2Self demonstrating superior capabilities in preserving both image fidelity and structural detail, while ZS-DeconvNet and Noise2Noise showed limitations under certain conditions.

### 4.1. Model Performance Across Modalities and Samples

A key finding of this study is the consistent performance of DIP, Noise2Void, and Self2Self across different imaging modalities—Rayleigh scattering, fluorescence, and Raman imaging—and varying sample conditions (both treated and untreated). These models consistently achieved high PSNR and SSIM values, indicating their robustness in noise suppression while retaining essential structural information.

In modality 1 (Table 1) (Laser: 785 nm, Rayleigh scattering, AOTF: 775 nm, Sample: Untreated 14C), DIP achieved the highest PSNR (41.83 dB) and FRC (0.994) values, indicating excellent noise reduction and preservation of high-frequency details critical for molecular imaging. Noise2Void performed similarly, with a PSNR of 38.96 dB and FRC of 0.997, demonstrating its ability to effectively handle different noise profiles without requiring paired data or external labels. These results are consistent with previous research highlighting the ability of these models to generalize well across various noisy datasets, especially in biomedical imaging [15,23].

Self2Self (Table 3), which uses dropout-based regularization, performed robustly with a PSNR of 39.15 dB and FRC of 0.994. Its adaptability to both treated and untreated samples made it an excellent choice for high-noise environments, where it maintained high-frequency information, crucial for subcellular structure preservation [34].

Similarly, for modality 2 (Table 4) (Laser: 660 nm, Fluorescence scattering, AOTF: 694 nm, Sample: Untreated 14C), the DIP, Noise2Void, and Self2Self models performed better than ZS-DeconvNet and Noise2Noise.

The performance of these three models (DIP, Noise2Void, and Self2Self) across both the treated 11B and untreated 11B samples showed remarkable consistency (Table 5 and Table 6) for Raman scattering using the 660 nm laser. In the treated 11B samples, where biomolecular markers and fluorescence signals were enhanced, the models excelled in noise suppression while preserving structural integrity, as reflected in SSIM values ranging from 0.90 to 0.99. In the untreated samples, which presented higher noise levels, Self2Self, DIP, and Noise2Void still maintained FRC values near 1.0, indicating their strong performance in retaining high-resolution details despite the more challenging noise conditions.

In contrast, ZS-DeconvNet and Noise2Noise showed significantly lower performance in the treated 11B and untreated 11B samples, where the absence of enhanced signals made noise suppression more difficult. ZS-DeconvNet, with a PSNR of 14.34 dB for the treated 11B samples and 14.43 dB for the untreated 11B samples and an FRC of 0.319 and −0.066, demonstrated strong smoothing but failed to retain fine structural details, particularly in the 11B-untreated Raman imaging. Noise2Noise performed little better than ZS-DeconvNet.

### 4.2. Generalization Across Modalities and Noise Profiles

An important observation from this study is the generalizability of DIP, Noise2Void, and Self2Self across all tested modalities and varying noise levels. These models demonstrated the ability to retain high-resolution structural features, as indicated by their consistently high FRC values (0.994–0.997), even in challenging conditions like Rayleigh scattering and untreated samples with weaker Raman signals. This is critical for multi-modal microscopy where noise characteristics vary significantly depending on both the imaging technique and sample type.

The self-supervised nature of these models—particularly Noise2Void and Self2Self—allowed them to adapt to diverse noise profiles without requiring paired training data, making them especially suitable for applications where labeled datasets are unavailable or difficult to generate [15,34]. In contrast, models like Noise2Noise, which rely on paired noisy data, struggled in real-world scenarios where such data are scarce.

The high PSNR values achieved by DIP and Noise2Void across all imaging conditions indicate that these models are well suited for tasks requiring high-fidelity restoration of subcellular structures. Their performance in untreated samples further highlights their potential for applications where noise levels are unpredictable, such as live-cell imaging or dynamic microscopy [18].

In summary, DIP, Noise2Void, and Self2Self proved to be the most robust across all modalities and noise conditions, making them excellent candidates for real-time imaging applications where maintaining high-resolution detail is paramount. Noise2Noise and ZS-DeconvNet, while effective in certain modalities, struggled with more complex noise profiles, limiting their broader applicability in multi-modal imaging tasks.

## 5. Conclusions

This study provides an in-depth comparative evaluation of five advanced zero-shot and self-supervised learning models—ZS-DeconvNet, Noise2Noise, Noise2Void, Deep Image Prior (DIP), and Self2Self—for denoising and super-resolution in multi-modal Raman light sheet microscopy applied to the visualization of 3D cell cultures. Across diverse modalities, including Rayleigh scattering, fluorescence, and Raman imaging, and across both treated and untreated samples, DIP, Noise2Void, and Self2Self consistently delivered the best results, achieving high PSNR, SSIM, and FRC values. DIP achieved the highest PSNR of >40 dB across different modalities and samples, demonstrating its unique ability to balance noise suppression with the preservation of fine structural details. Leveraging the inherent structure of convolutional neural networks as a prior, DIP operates in completely unsupervised manner, without requiring explicit training data, enabling adaptive regularization that excels in scenarios where labeled datasets are unavailable. Noise2Void and Self2Self provided similarly strong performance, with FRC values near 1.0, indicating their robustness in maintaining high-frequency structural information.

In contrast, ZS-DeconvNet and Noise2Noise showed limited effectiveness, particularly in high-noise environments, with ZS-DeconvNet using corrupted noise pairs within a zero-shot framework, struggled with the complex noise patterns characteristic of spheroid imaging, often resulting in oversmoothing and the loss of high-frequency details; similarly, Noise2Noise’s reliance on paired noisy datasets restricted its adaptability, highlighting the need for further optimization in these models. These findings reinforce the potential of self-supervised learning techniques, particularly in contexts where acquiring large labeled datasets is impractical.

The broader applicability of these methods to other imaging modalities, such as MRI, CT, and super-resolution microscopy, presents a promising direction for future research. Expanding these models to handle 3D volumetric data and exploring hybrid architectures could further enhance their utility in real-time biological imaging. Overall, this study demonstrates the versatility and effectiveness of self-supervised and zero-shot learning models for improving image quality in biomedical microscopy.

## Figures and Tables

**Table 1:** Imaging parameters for multi-modal acquisition.

Modality	Laser	Power	Exposure Time	AOTF Wavelength
Rayleigh (Modality 1)	785 nm	1 mW	100 ms	775 nm
	660 nm	1 mW	100 ms	650 nm
Fluorescence (Modality 2)	660 nm	130 mW	5000 ms	694 nm
Raman (Modality 3)	660 nm	130 mW	5000 ms	817 nm

**Table 2:** Selected parameters and hyperparameters of zero-shot and self-supervised algorithms for training.

Models	Learning Rate	Epochs	Optimizer	Loss Function	Batch Size
ZS-DeconvNet	0.001	100	Adam	MSE	1
Noise2Noise	0.001	100	Adam	MSE	1
Noise2Void	0.001	1000	Adam	MSE	1
DIP	0.001	1000	Adam	MSE	1
Self2Self	0.001	1000	Adam	MSE	1

**Table 3:** Quantitative comparison of zero-shot and self-supervised learning models using PSNR, SSIM, RMSE, and FRC metrics for modality 1 (Laser: 785 nm, Rayleigh scattering, AOTF: 775 nm, Sample: Untreated 14C).

Model	PSNR (dB)	SSIM	RMSE	FRC
ZS-DeconvNet	14.06	0.07	0.19	0.430
Noise2Noise	18.40	0.33	0.12	0.720
Noise2Void	38.96	0.96	0.01	0.997
DIP	41.83	0.95	0.008	0.994
Self2Self	39.15	0.91	0.01	0.994

**Table 4:** Quantitative comparison of zero-shot and self-supervised learning models using PSNR, SSIM, RMSE, and FRC metrics for modality 2 (Laser: 660 nm, Fluorescence scattering, AOTF: 694 nm, Sample: Untreated 14C).

Model	PSNR (dB)	SSIM	RMSE	FRC
ZS-DeconvNet	13.34	0.076	0.215	0.050
Noise2Noise	31.30	0.150	0.027	0.171
Noise2Void	44.60	0.828	0.006	0.924
DIP	44.74	0.810	0.006	0.936
Self2Self	41.52	0.735	0.008	0.889

**Table 5:** Quantitative comparison of zero-shot and self-supervised learning models using PSNR, SSIM, RMSE, and FRC metrics for modality 3 (Laser: 660 nm, Raman scattering, AOTF: 817 nm, Sample: Treated 11B).

Model	PSNR (dB)	SSIM	RMSE	FRC
ZS-DeconvNet	14.34	0.267	0.192	0.319
Noise2Noise	30.51	0.790	0.0298	0.592
Noise2Void	46.49	0.986	0.005	0.993
DIP	47.87	0.988	0.004	0.992
Self2Self	41.35	0.959	0.0085	0.980

**Table 6:** Quantitative comparison of zero-shot and self-supervised learning models using PSNR, SSIM, RMSE, and FRC metrics for modality 3 (Laser: 660 nm, Raman scattering, AOTF: 817 nm, Sample: Untreated 11B).

Model	PSNR (dB)	SSIM	RMSE	FRC
ZS-DeconvNet	14.43	0.290	0.189	−0.066
Noise2Noise	32.96	0.773	0.022	0.041
Noise2Void	48.43	0.991	0.004	0.934
DIP	50.62	0.993	0.003	0.928
Self2Self	40.81	0.954	0.009	0.836

## Data Availability

The data used to support the results of this study are included within the article. In addition, some of the data in this research are supported by the references mentioned in the manuscript. If you have any queries regarding the data, the data of this research is available from the corresponding author upon request.
